# Associations of Dietary Patterns With the Gut Microbiota in Older, Community-Dwelling Men

**DOI:** 10.1093/geroni/igaa057.3076

**Published:** 2020-12-16

**Authors:** James Shikany, Ryan Demmer, Abigail Johnson, Katie Meyer, Kristine Ensrud, Eric Orwoll, Deborah Kado, Lisa Langsetmo

**Affiliations:** 1 University of Alabama at Birmingham, Birmingham, Alabama, United States; 2 University of Minnesota, Minneapolis, Minnesota, United States; 3 University of North Carolina at Chapel Hill, Chapel Hill, North Carolina, United States; 4 Oregon Health & Science University, Portland, Oregon, United States; 5 University of California, San Diego, La Jolla, California, United States

## Abstract

We investigated associations of dietary patterns with composition and diversity of the gut bacterial microbiota in 517 community-dwelling older men (mean age 84.3 y) who were participants in the Osteoporotic Fractures in Men (MrOS) study. Eligible participants provided a stool sample and completed a food frequency questionnaire at the MrOS Visit 4 in 2014-2016. Dietary patterns were derived by factor analysis. 16S rRNA target gene sequencing was performed. Linear regression and PERMANOVA considered variation in alpha and beta-diversity by dietary pattern, and metagenomeSeq assessed taxonomic variation by dietary pattern. In multivariable-adjusted models, greater adherence to the Western pattern was positively associated certain taxa, including Alistipes, Desulfovibrio, Dorea, Eubacterium, and Ruminococcus, while greater adherence to the prudent pattern was positively associated with certain taxa, including Faecalibacterium, Lachnospira, and Paraprevotella. Dietary patterns were not associated with measures of alpha diversity; beta diversity measures were significantly associated with both Western and prudent patterns.

